# Development of an artificial intelligence system using deep learning to indicate anatomical landmarks during laparoscopic cholecystectomy

**DOI:** 10.1007/s00464-020-07548-x

**Published:** 2020-04-18

**Authors:** Tatsushi Tokuyasu, Yukio Iwashita, Yusuke Matsunobu, Toshiya Kamiyama, Makoto Ishikake, Seiichiro Sakaguchi, Kohei Ebe, Kazuhiro Tada, Yuichi Endo, Tsuyoshi Etoh, Makoto Nakashima, Masafumi Inomata

**Affiliations:** 1 Faculty of Information Engineering, Department of Information and Systems Engineering, Fukuoka Institute of Technology, 3-30-1 Wajiro-higashi, Higashi-ku, Fukuoka-City, Fukuoka, 811-0295 Japan; 2 Faculty of Medicine, Department of Gastroenterological and Pediatric Surgery, Oita University, 1-1 Idaigaoka, Hasama-machi, Yufu-City, Oita, 879-5593 Japan; 3 Customer Solutions Development, Platform Technology, Olympus Technologies Asia, Olympus Corporation, 2-3 Kuboyama-cho, Hachioji-City, Tokyo, 192-8512 Japan; 4 Faculty of Science and Technology, Division of Computer Science and Intelligent Systems, Oita University, 700 Dannoharu, Oita-City, Oita, 870-1192 Japan

**Keywords:** Artificial intelligence, Bile duct injury, Deep learning, Landmark, Laparoscopic cholecystectomy

## Abstract

**Background:**

The occurrence of bile duct injury (BDI) during laparoscopic cholecystectomy (LC) is an important medical issue. Expert surgeons prevent intraoperative BDI by identifying four landmarks. The present study aimed to develop a system that outlines these landmarks on endoscopic images in real time.

**Methods:**

An intraoperative landmark indication system was constructed using YOLOv3, which is an algorithm for object detection based on deep learning. The training datasets comprised approximately 2000 endoscopic images of the region of Calot's triangle in the gallbladder neck obtained from 76 videos of LC. The YOLOv3 learning model with the training datasets was applied to 23 videos of LC that were not used in training, to evaluate the estimation accuracy of the system to identify four landmarks: the cystic duct, common bile duct, lower edge of the left medial liver segment, and Rouviere’s sulcus. Additionally, we constructed a prototype and used it in a verification experiment in an operation for a patient with cholelithiasis.

**Results:**

The YOLOv3 learning model was quantitatively and subjectively evaluated in this study. The average precision values for each landmark were as follows: common bile duct: 0.320, cystic duct: 0.074, lower edge of the left medial liver segment: 0.314, and Rouviere’s sulcus: 0.101. The two expert surgeons involved in the annotation confirmed consensus regarding valid indications for each landmark in 22 of the 23 LC videos. In the verification experiment, the use of the intraoperative landmark indication system made the surgical team more aware of the landmarks.

**Conclusions:**

Intraoperative landmark indication successfully identified four landmarks during LC, which may help to reduce the incidence of BDI, and thus, increase the safety of LC. The novel system proposed in the present study may prevent BDI during LC in clinical practice.

Laparoscopic cholecystectomy (LC) is widely accepted worldwide [1]. LC is frequently performed by doctors who specialize in endoscopic surgery, and is considered an introductory level endoscopic surgery [2]. Currently, LC is the standard procedure for cholelithiasis and/or cholecystitis. Previous studies have described the standard procedure of LC [3], and the mechanism of bile duct injury (BDI) during LC [4]. The reported incidence of BDI during LC ranges from 0.2% to 1.1% [5–7], which is two to five times higher than during abdominal surgery.

The operative procedural steps involved in LC are described in Table 1 [2], and show the importance of identifying the cystic duct and the common bile duct to safely retrieve the gallbladder. Misidentifying the common bile duct as the cystic duct results in BDI [4, 8, 9]. A survey of over 600 surgeons in Japan, Korea, Taiwan, and the USA reported that 72.3% of all respondents experienced BDI or near-misses [10]; furthermore, 40.5% of respondents stated that BDI occurred because of misidentification of an anatomical landmark [11, 12]. These results suggest that there is a risk of BDI during LC regardless of the surgeon’s level of experience, and that identifying landmarks may prevent the BDI.

**Table 1:** Standard procedural steps followed during laparoscopic cholecystectomy

Step no	Procedure
1	Obtain the field of view by retracting the gallbladder (GB)
2	Confirmation of Calot’s triangle
3	Effective retraction of the GB to develop a plane in the Calot’s triangle area and identify its boundaries
4	Careful dissection to reveal the cystic artery and right hepatic artery
5	Confirmation of the running direction of common bile duct
6	Dissection around the cystic duct, and performance of the clipping method
7	Height of the cut-line of the cystic duct
8	Retraction of the GB to enable dissection of the GB from the GB bed with an adequate layer
9	Control the bleeding from the GB bed
10	Retrieve the GB

Figure 1 shows Calot's triangle in the gallbladder neck, with four landmarks exposed: the common bile duct, cystic duct, lower edge of the left medial liver segment, and Rouviere's sulcus. The use of these four landmarks to avoid BDI during LC has been introduced as the ‘critical view of safety’ method [13–16]. Considering the relatively high incidence of BDI in LC, it is doubtful whether the ‘critical view of safety’ technique is being effectively used in the operating room. The Society of American Gastrointestinal and Endoscopic Surgeons Safe Chole Task Force defines the critical view of safety as:

- The hepatocystic triangle is cleared of fat and fibrous tissue [17]. The hepatocystic triangle is defined as the triangle formed by the cystic duct, the common hepatic duct, and the inferior edge of the liver. The common bile duct and common hepatic duct do not have to be exposed.
- The lower one third of the gallbladder is separated from the liver to expose the cystic plate. The cystic plate is also known as the liver bed of the gallbladder and lies in the gallbladder fossa.
- Two and only two structures should be seen entering the gallbladder.

**Fig. 1:**
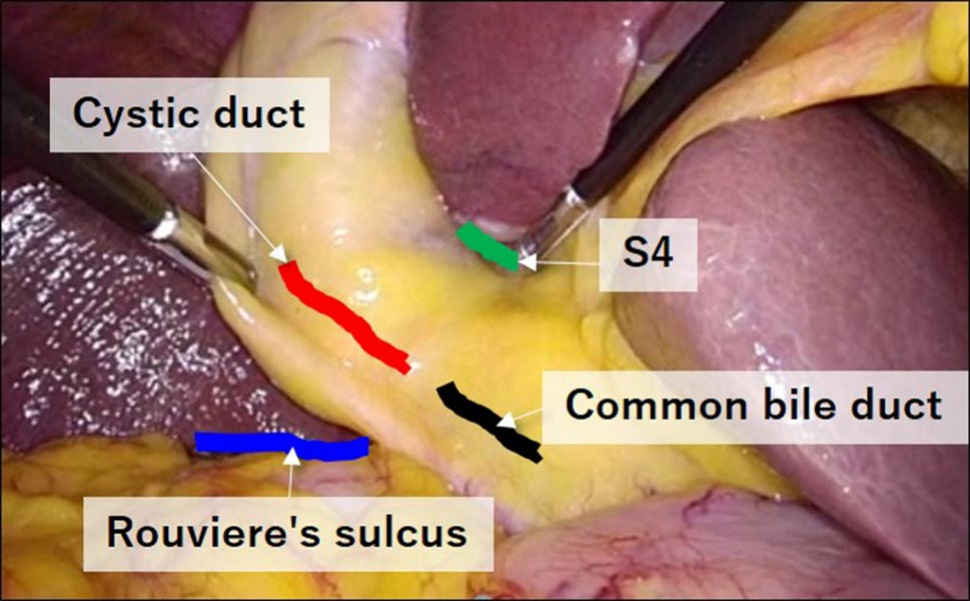
Photograph showing the anatomical landmarks in Calot's triangle: the common bile duct, cystic duct, lower edge of the left medial liver segment (S4), and Rouviere's sulcus

Intraoperatively, it is often difficult to accurately distinguish the common bile duct from the cystic duct, as these ducts are covered with fatty tissue. In such cases, surgeons should identify the lower edge of the left medial liver segment and Rouviere's sulcus. Assuming a straight line connecting Rouviere's sulcus and the lower edge of the left medial liver segment, the liver bed is basically ventral to this straight line, so both are important landmarks to prevent BDI. In addition, Rouviere’s sulcus is a useful anatomical landmark for beginning dissection of Calot’s triangle [18, 19]. For these reasons, in this study, we added Rouviere’s sulcus as the fourth landmark.

To consciously check each landmark, surgeons require a system that can accurately identify the landmarks. Thus, we devised an artificial intelligence system that intraoperatively indicates the location of the four landmarks to aid in preventing BDI.

The present article describes the technological components necessary to detect the four landmarks, and discusses the results of a verification experiment that implemented these technologies.

## Materials and methods

### Preparation of datasets

We used an algorithm of real-time object detection based on deep learning to realize intraoperative landmark indication on endoscopic camera images. Two-hundred and thirty videos of LC performed in Oita University were obtained. As the degree of difficulty in LC increases in tandem with the extent of fibrosis and/or scarring inside the abdominal cavity, the technical platform of our system was established using videos with minimal fibrosis and/or scarring; videos with bleeding or less-visible landmarks were also excluded. From the remaining 99 videos, the scenes showing Calot's triangle in the gallbladder neck were extracted and saved in MP4 data format; these short videos were assigned sequential numbers.

The following steps were repeatedly implemented to maximize the effectiveness of the creation and learning of the datasets: (i) a short video was divided into still image files, (ii) the first still image was selected for labeling, (iii) the similarity between the previously selected image and the subsequent image was calculated, and finally (iv) the images with a degree of similarity that exceeded a certain threshold were selected for landmark labeling. Using these processes made it possible to reduce the number of datasets and avoid overlapping the emerging pattern of the landmarks.

**Fig. 2:**
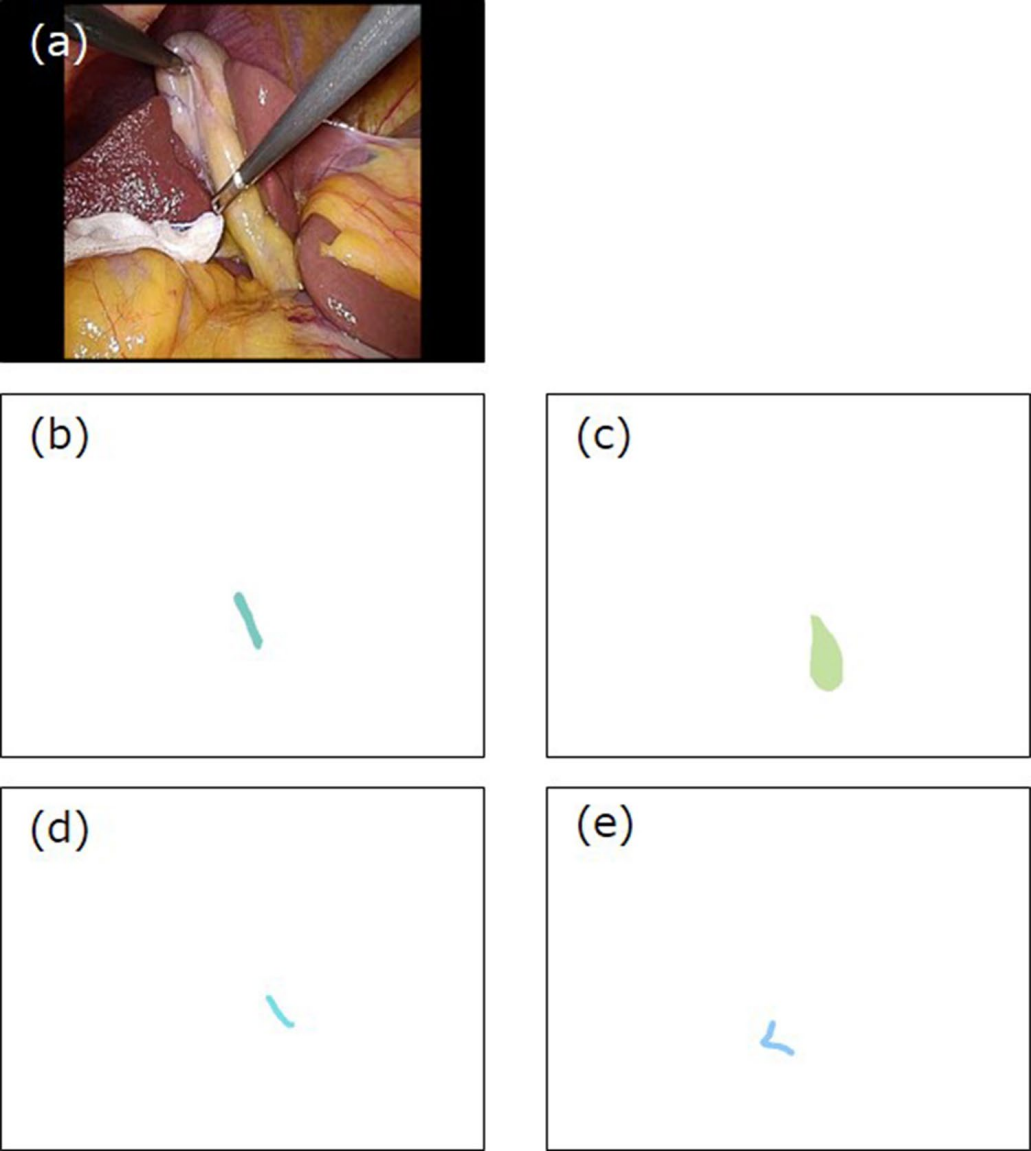
Example of a dataset for deep learning. **A** endoscopic image, **B** common bile duct, **C** cystic duct, **D** lower edge of the left medial liver segment (S4), **E** Rouviere's sulcus

All short videos were used to create the datasets for the deep learning training and for the evaluation of estimation accuracy of the training model, in which expert surgeons labeled the areas containing each landmark on the endoscopic images. One dataset constituted five images: the image of the endoscopic camera, the common bile duct, cystic duct, lower edge of the left medial liver segment, and Rouviere’s sulcus (Fig. 2); however, a landmark may not be visible depending on an organ’s location and/or the severity of inflammation. These images were saved in PNG data format.

To maintain a high degree of accuracy of the datasets, labeling was performed only by the two expert surgeons who had experienced over 200 LC procedures. We confirmed subjectively that concordance between the expert surgeons was poor. Therefore, we re-evaluated the annotation datasets in which the two expert surgeons first shared the videos, to efficiently create the datasets, and performed a final check of the datasets that they labeled, to complete the annotation data. We assumed that using at least these methodologies for annotation can eliminate annotation data that are clearly wrong. Eventually, the expert surgeons labeled the evaluated structures against 2339 images of the endoscopic camera and labeled 2119 images of the common bile duct, 1895 images of the cystic duct, 2144 images of the lower edge of the left medial liver segment, and 2012 images of Rouviere’s sulcus. The number of datasets prepared in this study was 2339, and we augmented the datasets 26 times to train the YOLOv3 learning model. Of the 99 videos, 76 videos were used to train the deep learning model, and 23 videos were used to evaluate the estimation accuracy of the deep learning model created with the training datasets.

### Detection of landmarks

To intraoperatively detect and indicate the locations of the landmarks on an endoscopic image, both high-accuracy detection and high-speed computation were required. In the research field of image recognition algorithms based on deep learning, various methods have been proposed to identify the position and the class of an object [20, 21]. In the present study, we used YOLOv3 [22], as this algorithm is reportedly superior to other algorithms regarding computing speed and class discrimination accuracy.

**Fig. 3:**
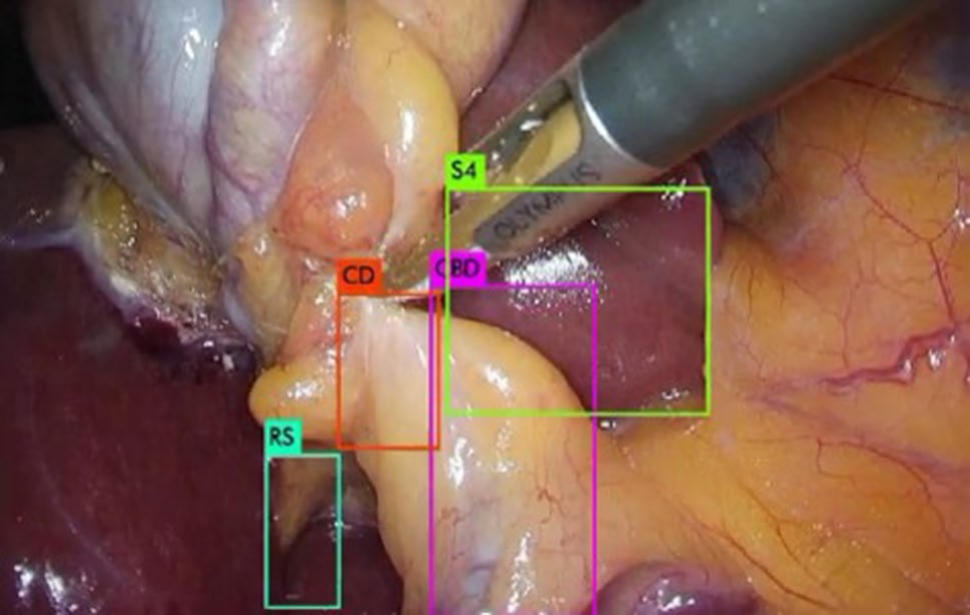
Bounding boxes for each landmark as an output image of YOLOv3. CD: cystic duct; CBD: common bile duct; S4: lower edge of the left medial segment; RS: Rouviere’s sulcus

The source code for YOLOv3 was downloaded from the developer's website [22]. Figure 3 shows an example of an output result of YOLOv3, where the colored bounding boxes show the respective position and class of each landmark.

### Prototype development

The prototype was composed of an endoscopic camera (OLYMPUS LTF-S190-10; Olympus Corp., Tokyo, Japan), a video processor (VISERA ELITE II; Olympus Corp.), and a desktop computer. The computer had one Graphics Processing Unit (Tesla V100; NVIDIA Corp., Santa Clara, CA) mounted for the calculation of the YOLOv3 learning model, and an image conversion board installed to load the output signal from the endoscopic camera. The YOLOv3 learning model was installed in the computer and used to calculate the coordinates of the bounding box for each landmark. The video processer displayed the endoscopic image in which the bounding boxes were overlaid on the monitors in the operating room.

### Evaluation of the learning model

The detection accuracy and computation time capabilities of YOLOv3 have already been quantitatively evaluated [22]. Thus, the estimation accuracy of the YOLOv3 learning model depends on the ability to accurately describe the bounding boxes on the endoscopic image, and on the ability of the expert surgeons to create the datasets. The estimation accuracy was defined as the quantitative index of the ability to indicate the location of the landmark. We performed both quantitative and subjective evaluations using the annotation data, which was not used in constructing the YOLOv3 learning model. In this study, we applied the YOLOv3 learning model to the 23 short videos to evaluate the landmark estimation accuracy. Next, we created new video files in which we overlaid bounding boxes for each landmark on the endoscopic image. The average computation speed required to draw the bounding box on an endoscopic image was 37.2 frames per second.

Generally, the augmentation of training datasets is recommended to improve the performance of deep learning [23]. As the appearance of the abdominal organs differs between patients, and there are individual differences in the skill of each endoscopic operator, we used the "ImageDataGenerator class" in Keras [24], which is a network library used to augment training datasets; the number of augmentations was 26, with the following parameters: rotation range, 30.0; shear range, 0.4; and zoom range, 0.4. Additionally, the contrast of the image of the endoscopic camera was increased to 0.2–10.0 with respect to the original image.

### Development of a prototype for the verification experiment

**Fig. 4:**
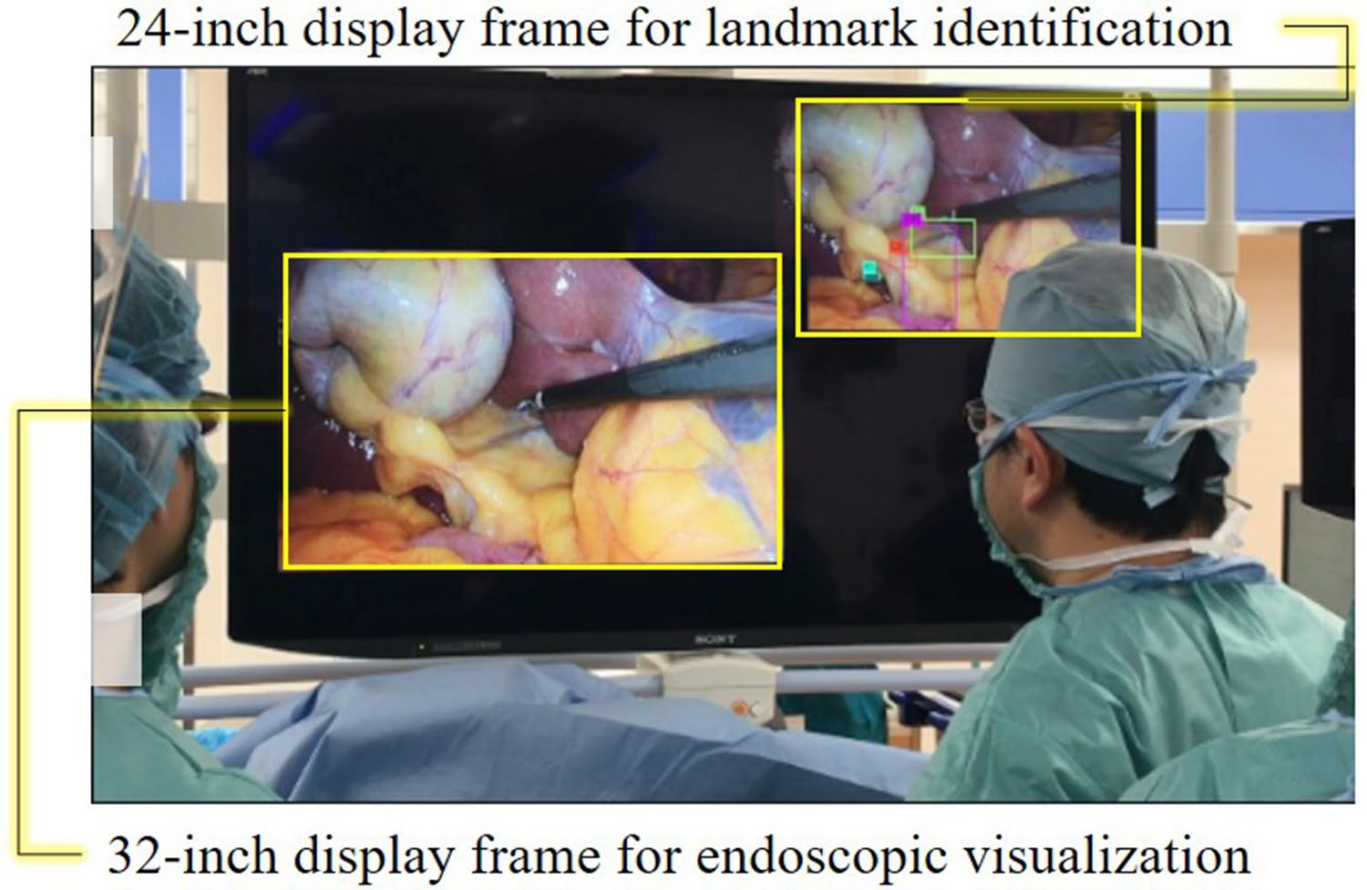
The layout of the main monitor screen during the verification experiment

The prototype was connected to an integrative system (EndoALPHA; Olympus Corp.) that made it possible to draw a display that indicated the locations of the landmarks on the monitors in the operating room. As shown in Fig. 4, the displays for the landmark indications and the endoscopic images were shown on a 50-inch 8 K monitor (LMD-X550ST; Sony Corp., Tokyo, Japan).

## Results

### Landmark estimation accuracy

The YOLOv3 learning model was trained with the augmented datasets applied to the 23 short videos prepared for the performance evaluation. We applied the YOLOv3 learning model to the 23 annotation datasets that were not used in training. The annotation datasets for the evaluation constituted 190 images of the common bile duct, 186 images of the cystic duct, 192 images of the lower edge of the left medial liver segment, and 190 images of Rouviere’s sulcus, and all images were labeled against 194 images of the endoscopic camera. The objective evaluation using average precision resulted in low values, with the average precision of the YOLOv3 learning model for each landmark computed as follows: common bile duct: 0.320, cystic duct: 0.074, lower edge of the left medial liver segment: 0.314, and Rouviere’s sulcus: 0.101.

We confirmed that the YOLOv3 learning model was able to outline the bounding boxes on each landmark against the video files. Consequently, the two expert surgeons who made the annotation datasets, subjectively judged whether the video file provided the information required to prevent the occurrence of BDI during LC, based on consensus. Table 2 shows the estimation accuracy as assessed by the expert surgeons for the YOLOv3 learning model. Although some cystic ducts were not detected, 22 of the 23 videos were judged to have good landmark identification.

**Table 2:** Results of the estimation accuracy evaluation of the YOLOv3 learning model trained with our datasets

Video no	CBD	CD	S4	RS	Overall Judgment
1	Ο	Ο	Ο	Ο	Ο
2	Ο	Ο	Ο	**OS**	Ο
3	Ο	Ο	Ο	Ο	Ο
4	Ο	Ο	Ο	Ο	Ο
5	Ο	Ο	Ο	Ο	Ο
6	Ο	**×**	Ο	Ο	Ο
7	Ο	**×**	Ο	Ο	Ο
8	Ο	Ο	Ο	**OS**	Ο
9	Ο	Ο	Ο	Ο	Ο
10	Ο	**×**	Ο	Ο	Ο
11	**×**	**×**	Ο	**×**	**×**
12	Ο	Ο	Ο	Ο	Ο
13	Ο	**×**	Ο	Ο	Ο
14	Ο	Ο	Ο	Ο	Ο
15	Ο	Ο	Ο	Ο	Ο
16	Ο	Ο	Ο	Ο	Ο
17	Ο	**×**	Ο	Ο	Ο
18	Ο	Ο	Ο	Ο	Ο
19	Ο	Ο	Ο	Ο	Ο
20	Ο	Ο	Ο	Ο	Ο
21	Ο	Ο	Ο	Ο	Ο
22	Ο	Ο	Ο	Ο	Ο
23	Ο	Ο	Ο	Ο	Ο

*CBD* common bile duct; *CD* cystic duct; *S4* lower edge of the left medial segment; *RS* Rouviere’s sulcus; *OS* out of sight (i.e., not visualized)

### Verification experiment

To verify the clinical significance of our proposed system in an operating room, we used the prototype landmark indication system in a verification experiment using images from LC performed in an 82-year-old woman with cholelithiasis. This portion of the study was approved by the ethics committee of Oita University, and the patient provided informed consent. As shown in Fig. 4, the display frames for landmark indication and the endoscopic image were located separately on the main monitor screen, so we assumed that there was no effect on the progress of the operation. The impressions of the surgeons are described in the discussion.

## Discussion

In this study, we constructed a learning model based on YOLOv3 to detect four anatomical landmarks during LC. Although the average precision for each landmark was poor, the two surgeons involved in the annotation agreed that the YOLOv3 learning model successfully indicated the landmarks essential to avoid BDI in 22 of the 23 videos. The results subjectively evaluated by the two surgeons indicated that the YOLOv3 learning model can flexibly deal with individual patient differences related to well-known variations in biliary anatomy [25]. We actually excluded videos in which we confirmed bleeding, high fibrosis, and scarring in the endoscopic images, and did not consider variations in biliary anatomy. The reason that video number 11 failed was assumed to be because the areas showing the landmarks were in the lower part of the endoscopic image. In addition, the shift toward the vertical direction was not included in the data augmentation parameters. Data augmentation contributes greatly to deep leaning, but we believe that data augmentation must be applied to medical images with great care because this method generates non-existent images. Although we aimed to improve the accuracy of the YOLOv3 learning model, in this study, we chose to use landmarks important in the actual surgery, and we made certain decisions; for example, rotation was allowed but inversion was not allowed.

Ideally, it is desirable that a landmark indication system accommodates all patients under consideration and can be used in daily clinical practice. However, we believe that the number of videos and the parameters for data augmentation necessary for the system to satisfy this requirement depend on the purpose of the system. In this study, we intentionally excluded videos in which bleeding, high fibrosis, and scarring interfered with the visibility of the landmarks, and the YOLOv3 learning model then successfully indicated the landmarks with high accuracy. To use this system with more difficult cases, it is necessary to prepare a video with a corresponding degree of difficulty.

The two expert surgeons in this study performed the annotation and confirmed that the YOLOv3 learning model performed well when rendering the bounding boxes for each landmark. However, the surgeons may have made mistakes secondary to errors in human visual perception. Way et al. demonstrated that 97% of the causes of BDI were secondary to errors in human visual perception and stated that the most effective strategy for overcoming these types of errors is the evolution of technology [26]. Artificial intelligence is a technology that can improve its performance depending on the amount and quality of annotation data. Evaluating annotation data with multiple expert surgeons can eliminate data errors secondary to human visual perception; therefore, a complete AI system that helps surgeons avoid making incorrect intraoperative decisions is expected in the near future.

We successfully used a prototype of the landmark indication system during a verification experiment in a patient with cholelithiasis, in this study. A major improvement in detection accuracy was confirmed when the surgeon optimized the visibility of the landmarks. This suggests that experienced surgeons implicitly expand the operation field to make the landmarks more obvious, as the datasets used in the training of YOLOv3 were based on the images of LC performed by experienced surgeons. Through the verification experiment, we confirmed the clinical significance of the proposed system, and identified issues that require resolution to optimize the outcomes. The main issue was the flickering of the bounding boxes caused by the continuity of YOLOv3 in detecting the landmarks; this flickering can be reduced using a filtering technique for the coordinates of the bounding boxes.

The goal of the present study was to achieve favorable outcomes using an artificial intelligence system that detected four landmarks during LC. This system was developed to reduce the incidence of BDI during LC, as one of the major causes of BDI is misidentifying the cystic duct as the common bile duct and/or hepatic duct; this is called “classic laparoscopic injury,” and many investigators have analyzed its mechanism [4, 9, 10, 27].

Currently, the most effective precautionary measure for preventing BDI or near-miss BDI secondary to misidentifying the cystic duct during LC is advice from a member of the surgical team other than the operator [10]. The risk of BDI is reportedly lower in hospitals with a surgical residency program, which highlights the importance of constantly raising the awareness of potential BDI through surgical education [28]. In the future, landmark indication using artificial intelligence may become an important tool that increases the safety of LC.

As well as increasing the safety of LC, easy and accurate landmark detection streamlines the operation. In laparoscopic surgery, surgeons generally rely on visual information because of the lack of tactile sensation. Thus, the surgeon needs to use knowledge based on their own surgical experience and the anatomical position of the organ to recognize the landmarks.

The verification experiment in this study showed that the landmark indication system we described yielded a similar benefit to having an expert surgeon in the surgical team (Fig. 4). The empirical value of expert surgeons is clearly related to the outcome of therapy, as high-volume centers achieve better outcomes regarding surgical time, bleeding volume, and postoperative complication rates compared with other institutions [29, 30].

The proposed intraoperative landmark indication system uses an artificial intelligence technique intraoperatively. The artificial intelligence technique has already been applied in preoperative diagnosis via the detection of abnormalities on computed tomography and radiographic images [31]. Artificial intelligence has also been used for automatic segmentation of the heart and measuring the aorta [32]. Furthermore, the capability of artificial intelligence to detect stomach cancer and polyps during endoscopic inspection is equal to that of skilled doctors [33, 34], and is beginning to be used in clinical practice. Thus, the use of artificial intelligence can effectively share the empirical value of expert surgeons, which improves the outcome of therapy. The landmark indication system proposed in the present study may aid in laparoscopic surgery in other fields, such as gastrointestinal and colorectal surgeries. However, there are no precedents for the use of a medical system based on artificial intelligence for intraoperative decision-making, and the advantages require clarification in the clinical setting.

Guidelines for the use of artificial intelligence in medicine have not yet been established, and it is difficult to clearly understand how deep learning is used to make judgments. It is a common misconception that all artificial intelligence systems automatically change their characteristics during use. In future, the utility of the landmark indication system will be improved by increasing the number of datasets. In addition, a clinical performance test will be scheduled in the near future.

## Conclusions

We proposed an intraoperative landmark indication system to prevent BDI in LC. Although the average precisions for each landmark in the YOLOv3 learning model trained with our datasets were low, the two surgeons agreed that valid indications of the landmarks were confirmed in 22 of the 23 LC videos and the prototype system was successfully used in a verification experiment. The use of intraoperative landmark indication systems will help reduce the incidence of BDI, and will increase the safety of LC.
